# The rs2910164 Genetic Variant of miR-146a-3p Is Associated with Increased Overall Mortality in Patients with Follicular Variant Papillary Thyroid Carcinoma

**DOI:** 10.3390/ijms19030655

**Published:** 2018-02-26

**Authors:** Marta Kotlarek, Anna Kubiak, Małgorzata Czetwertyńska, Michał Świerniak, Wojciech Gierlikowski, Monika Kolanowska, Elwira Bakuła-Zalewska, Sissy M. Jhiang, Krystian Jażdżewski, Anna Wójcicka

**Affiliations:** 1 Genomic Medicine, Medical University of Warsaw, 02-097 Warsaw, Poland; mkotlarek@wum.edu.pl (M.K.); a.kubiak@cent.uw.edu.pl (A.K.); m.swierniak@cent.uw.edu.pl (M.Ś.); wgierlikowski@wum.edu.pl (W.G.); m.kolanowska@cent.uw.edu.pl (M.K.); k.jazdzewski@cent.uw.edu.pl (K.J.); 2 Centre of New Technologies, University of Warsaw, 02-097 Warsaw, Poland; 3 Department of Nuclear Medicine & Endocrine Oncology, Maria Sklodowska-Curie Memorial Cancer Center and Institute of Oncology, 02-781 Warsaw, Poland; malgorzata.czetwertynska@coi.pl; 4 Department of Pathology, Maria Sklodowska-Curie Memorial Cancer Center and Institute of Oncology, 02-781 Warsaw, Poland; elwirabz@onet.eu; 5 Department of Physiology and Cell Biology, The Ohio State University, Columbus, OH 43210, USA; jhiang.1@osu.edu

**Keywords:** microRNA, miRNA-146a, rs2910164, papillary thyroid carcinoma (PTC), thyroid carcinoma

## Abstract

Aberrant expression of the sodium-iodide symporter (NIS) and the resistance to post-operative radioactive iodide treatment is a crucial cause of higher mortality of some thyroid cancer patients. In this study, we analyzed the impact of miR-146a on the expression and function of NIS and on the overall survival of thyroid cancer patients. The study included 2441 patients (2163 women; 278 men); including 359 cases with follicular variant of papillary thyroid carcinoma (fvPTC). miR:*NIS* interactions were analyzed in cell lines using in vivo binding and inhibition assays and radioactive iodine uptake assays. Tumor/blood DNA was used for rs2910164 genotyping. Overall survival was assessed retrospectively. In the results, we showed that miR-146a-3p directly binds to and inhibits *NIS*. Inhibition of miR-146a-3p restores the expression and function of *NIS,* increasing radioactive iodine uptake. Rs2910164 functional variant within miR-146a-3p is associated with increased overall mortality among fvPTC female patients. The deaths per 1000 person-years were 29.7 in CC carriers vs. 5.08 in GG/GC-carriers (HR = 6.21, *p = 0.006*). Higher mortality of CC vs. GG/GC carriers was also observed in patients with lower clinical stage (HR = 22.72, *p* < 0.001), smaller tumor size (pT1/pT2) (HR = 25.05, *p < 0.001*), lack of extrathyroidal invasion (HR = 9.03, *p* = 0.02), lack of nodular invasion (HR = 7.84, *p = 0.002*), lack of metastases (HR = 6.5, *p* = 0.005) and older (age at diagnosis >50 years) (HR = 7.8, *p = 0.002*). MiR-146a-3p underwent somatic mutations in 16.1% of analyzed specimens, mainly towards the deleterious C allele. In this report we propose a novel molecular marker of the clinical outcome of fvPTC patients. Rs2910164 increases the overall mortality with inhibition of NIS and disruption of radioiodine uptake as a possible mechanism.

## 1. Introduction

Thyroid cancer is the most common endocrine malignancy, highly heterogeneous with respect to its molecular and clinical properties. Papillary thyroid carcinoma (PTC) constitutes close to 85% of thyroid cancer cases. Although PTC is considered a disease of low mortality, as 90% of patients present with a 10-year disease free survival, a significant number of patients die within few years from the disease onset. There is an urgent need for markers allowing for stratification of low and high-risk patients. Recent data show that such stratification can be performed based on molecular markers, such as a germline polymorphism rs966423 in *DIRC3*, whose presence significantly increases the overall mortality of PTC patients [1]. Apart from germinal genetic variants, tumor BRAF V600E mutation is associated with cancer-related mortality [2].

Clinically, one of the crucial reasons for higher mortality of some thyroid cancer patients is their resistance to 131-I treatment in course of reduced radioidine uptake due to absence/decrease of the sodium-iodide symporter NIS expression [3]. Two recent studies have shown the expression of *NIS* is regulated by microRNA-146b [4,5]. MicroRNAs (miRs) are small RNAs of an average length of 20–22 nucleotides that regulate the expression of protein-coding genes by annealing to specific target sequences in the 3′untranslated regions (3′UTRs) of their transcripts [6]. MicroRNA-146a is almost identical counterpart of miR-146b, and is a known oncomiR involved in thyroid tumorigenesis [7,8]. The rs2910164 in miR-146a-3p is a well-studied example of a miR polymorphism (miR-SNP) which alters both the expression level of the miR, and the target specificity of the miR [9]. Functional studies showed that the presence of the “C” allele results in reduced amount of both mature miR-146a-5p and miR-146a-3p when compared to the sequence with “G” allele, and all the mature forms of miRNA-146a have profoundly different target genes [10]. The transcriptome analysis of PTC tumors with the GG and GC genotype in miR-146a-3p revealed a significant difference in the expression of genes implicated in apoptosis, cell differentiation and blood vessel development, often involved in the process of tumorigenesis [11]. Further studies have shown the association of the SNP with the risk for thyroid cancer in Caucasian populations [9,12], but the results in other populations were conflicting [13,14].

Here, we show that the G>C polymorphism (rs2910164) in miR-146a-3p is associated with increased overall mortality among patients with follicular variant PTC. We also show that miR-146a-3p directly binds the 3′UTR of NIS, and NIS expression is increased by miR-146a-3p inhibition. Interestingly, miR-146a-3p underwent somatic mutation in cancer tissues with 70% of mutations directed towards the “C” allele. Taken together, the association of rs2910164 with increased mortality among patients with follicular variant PTC may involve the regulation of *NIS* gene with an impact on tumor radioiodine uptake.

## 2. Results

### 2.1. The rs2910164 Genetic Variant of miR-146a-3p Is Associated with Increased Mortality in Patients with Follicular Variant PTC

The overall mortality of all PTC patients was 5.5% (135/2441). There were 113 deaths among 2082 patients with conventional PTC (5.4%) and 22 deaths among 359 fvPTC patients (6.1%) (Table S1). Rs2910164 polymorphism is associated with increased mortality among female patients with fvPTC (*p* = 0.006). The deaths per 1000 person-years were 29.7 (95% CI, 3.6–107.3) in CC carriers vs. 5.08 (95% CI, 2.96–8.13) in GG/GC-carriers; the hazard ratio (HR) was 7.03 (95% CI, 1.58–31.19; *p* = 0.003). After adjustment for age the HR was 6.21 (95% CI, 1.38–27.93; *p* = 0.006). MiR-146a-CC was not associated with increased mortality among patients with conventional PTC (Table S1).

**Figure 1:**
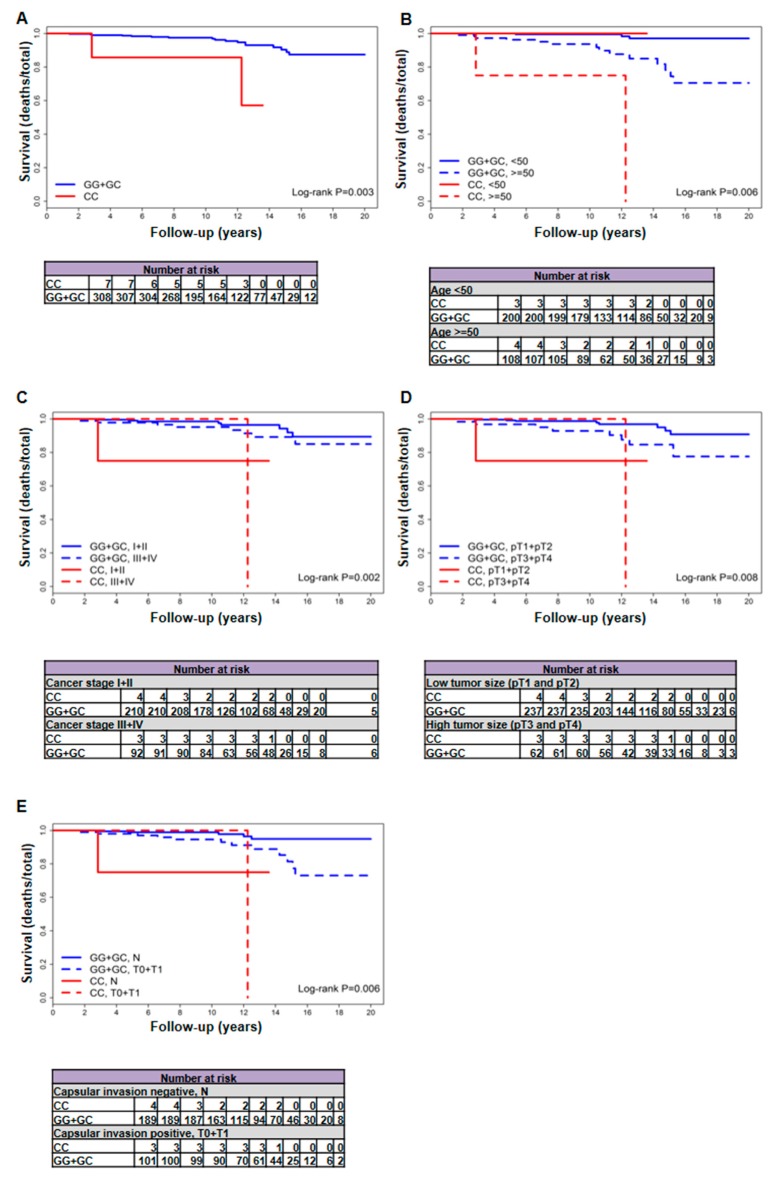
Interaction between rs2910164 and clinical factors in shaping the overall mortality Kaplan–Meier survival curves of the interaction of rs2910164 variant with the overall survival (**A**) and the clinicopathological risk factors: age at diagnosis (**B**), clinical cancer stage (**C**), tumor size (**D**) or capsular invasion (**E**) in patients with follicular variant of papillary thyroid carcinoma. In all panels, follow-up time is truncated at 20 years. In each panel, *p* values are from the log-rank test.

In female patients with fvPTC, rs2910164 modifies the mortality risk associated with several clinicopathological risk factors, including extrathyroidal invasion, and distant metastases (Table 1). The increased mortality was observed in patients generally considered “low-risk”, i.e., with a low clinical stage of the disease (stage I and II), small tumor size (pT1 and pT2), no extrathyroidal invasion, and no distant metastases. Importantly, the hazard ratios (HRs) remained statistically significant after adjustment for age. The differences in overall mortality in patients with the rs2910164 risk genotype and clinical cancer stage, tumor size or capsular invasion were reflected in the Kaplan-Meier survival curves (Figure 1).

In patients with lower clinical cancer stage (stage I and II) of fvPTC, the deaths per 1000 person-years were 29.27 (95% CI, 0.74–163.06) vs. 3.66 (95% CI, (1.58–7.22) in CC vs. GG/GC patients (adjusted HR, 22.72 (95% CI, 2.04–253.66); (logrank *p* < 0.001)) in contrast to 7.5 (95% CI, 3.24–14.77) in patients with higher cancer stage and GG/GC genotype (Table 1). In patients with smaller tumor size (pT1 and pT2) of fvPTC, the deaths per 1000 person-years were 29.27 (95% CI, 0.74–163.06) vs. 3.23 (95% CI, 1.39–6.36) in CC vs. GG/GC patients (adjusted HR, 25.05 (95% CI, 2.23–280.9); (logrank *p* < 0.001)) in contrast to 11.36 (95% CI, 4.9–22.38) in patients with higher tumor size and GG/GC genotype (Table 1). In patients with no extrathyroidal invasion, deaths per 1000 person-years were 29.27 (95% CI, 0.74–163.06) vs. 2.48 (95% CI, 0.81–5.79) in CC vs. GG/GC patients (adjusted HR, 9.03 (95% CI, 0.94–87.18); (logrank *p* = 0.021)) in contrast to 10.75 (95% CI, 5.55–18.77) in extrathyroidal invasion-positive GG/GC patients (Table 1). In patients with no distant metastases, deaths per 1000 person-years were 29.7 (95% CI, 3.6–107.3) vs. 5.12 (95% CI, 2.93–8.32) in CC vs. GG/GC patients (adjusted HR, 6.5 (95% CI, 1.43–29.52); (logrank *p* = 0.005)) in contrast to 11.17 (95% CI, 0.28–62.25) in distant metastases-positive GG/GC patients (Table 1). In patients older than 50 years of age, deaths per 1000 person-years were 67.05 (95% CI, 8.12–242.2) vs. 12.64 (95% CI, 6.91–21.22) in CC vs. GG/GC patients (adjusted HR, 7.85 (95% CI, 1.68–36.55); (logrank *p* = 0.002)) in contrast to 1.34 (95% CI, 0.28–3.91) in GG/GC patients younger than 50 years of age (Table 1).

### 2.2. MiR-146a-3p Directly Binds the 3′UTR of NIS

**Figure 2:**
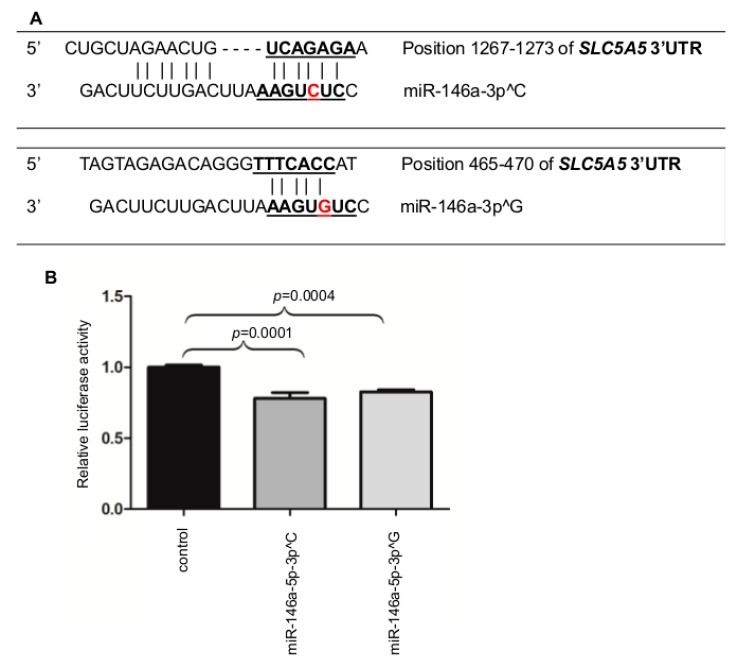
Positions of the putative miRNA binding sites in 3’UTRs of *SLC5A5*, identified by TargetScan and miRanda. Nucleotides of perfect complementarity are shown as a seed match (**A**) and the rs2910164 is indicated in red. (**B**) The effect of miR-146a-5p-3p^C or miR-146a-5p-3p^G on the luciferase activity mediated from the *NIS* 3′UTR is shown relative to control (cells transfected with control miR). The results are normalized to *Renilla* luciferase and derived from three experiments, each performed in triplicates. Binding of microRNAs to the 3′UTR of *NIS* resulted in a significant reduction in the luciferase activity for miR-146a-5p-3p^C (22%, *p* = 0.0001) and miR-146a-5p-3p^G (17%, *p* = 0.0004). The graphs show the mean, along with deviations from mean (SEM). Statistical analysis was performed using an unpaired *t* test.

The rs2910164 resides in the seed region of miR-146a-3p and alters the functioning of the mature miRs produced from this arm. We performed in silico analysis to identify target genes for the two allelic variants of miR-146a-3p, and identified *NIS* as the putative target for both products of a heterozygous gene: miR-146a-3p^C and miR-146a-3p^G; two alternative binding sites are broadly conserved among vertebrates (Figure 2A). We found no binding site for miR-146a-5p produced from the other arm of pre-microRNA-146a.

To analyze the direct interaction between the microRNAs and *NIS*, HeLa cell line with low endogenous expression of all the analyzed genes was transfected with a microRNA expression plasmid (miR-146a-5p-3p^C or miR-146a-5p-3p^G) and a plasmid harboring the 3’UTR sequence of *NIS*. Direct interaction between the genes was determined in luciferase assay. A significant reduction of luciferase activity driven from the *NIS* 3’UTR was demonstrated for miR-146a-5p-3p^C (22%, *p* = 0.0001), and miR-146a-5p-3p^G (17%, *p* = 0.0004) (Figure 2B).

### 2.3. Inhibition of the miR-146a-3p Restores the Expression and Function of NIS

**Figure 3:**
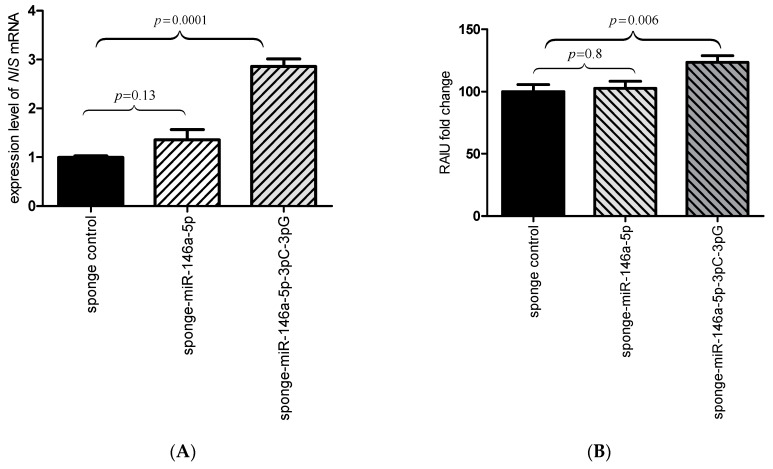
Inhibition of the miR-146a-5p and miR-146a-3p results in upregulation of *NIS* (sodium-iodide symporter) and increased radioactive iodine uptake. Transfection of the tRA/H treated MCF7 cell line with a sponge miRs and resulting inhibition of both variants of miR-146a-3p, but not miR-146a-5p, leads to a significant increase in *NIS* mRNA levels (2.86 fold, *p* = 0.0001). (**A**) and radioactive iodine uptake, RAIU (1.24 fold, *p* = 0.006); (**B**). the graphs show the mean, along with deviations from mean (SEM). Statistical analysis was performed using an unpaired *t* test.

Inhibition of microRNAs was attained with the use of specific microRNA sponge plasmids introduced to tRA/H-treated MCF7 cell line that expresses the *NIS* gene at high level. The use of microRNA sponges resulted in a significant increase of *NIS* mRNA levels for sponge-miR-146a-5p-3p^C-3p^G (2.86 fold, *p* = 0.0001), while no significant increase of NIS mRNA levels was seen for sponge-miR-146a-5p (*p* = 0.13) (Figure 3A). The functional consequence of increased *NIS* expression via sponge-miR-146a-5p-3p^C-3p^G was verified by increased RAIU (1.24-fold, *p* = 0.006). In contrast, sponge miR-146a-5p did not increase NIS mRNA level nor RAIU (Figure 3B). This result confirms that the *NIS*:miR-146a regulatory interaction is mediated by the polymorphic miR-146a-3p (miR-146a-3p^C and miR-146a-3p^G), while miR-146a-5p has no effect on *NIS*.

### 2.4. The miR-146a-3p Undergoes Somatic Mutations in Cancer Tissue

The “C” allele in miR-146a-3p influences the overall mortality of fvPTC patients, but the germline CC homozygosity occurs very rarely. Thus, we analyzed the miR-146a-3p status in cancer tissue samples. The pairs of cancer tissue DNA and germline DNA derived from the same patient were available for 186 patients, including 157 conventional PTC and 29 fvPTC. Genotyping of rs2910164 revealed that the miR undergoes somatic mutation in 30 (16.1%) of analyzed specimens, including both conventional (28 cases) and fvPTC (2 cases), and 70% of all mutations (21/30) were towards the “C” allele (Table S3). Also, 26% (8/30) of the mutations resulted in homozygosity for the “C” allele. Unfortunately, the tissue sample number was not sufficient to reveal significant association with the clinical outcome of the analyzed patients.

## 3. Discussion

The uniqueness of genetic variations in microRNA sequences (miR-SNPs) arises from the fact that they might influence both the expression levels and regulatory specificity of a miRNA [15]. The rs2910164 in miR-146a-3p is a well-documented polymorphism affecting both the expression and functionality of mature microRNAs expressed from the miR-146a precursor [9,10,11]. In this study we have shown that both miRs expressed from the miR-146a precursor regulate the expression of sodium-iodide transporter (we did not show that C vs G allele modulate NIS expression differently). It was previously shown that miR-146a-5p regulates the expression of several thyroid-related genes, such as the thyroid hormone receptor beta (*THRB*) [8]. Since both *NIS* and *THRB* genes are essential for the synthesis of thyroid hormones, miR-146a can be proposed as an important regulator of this process.

We have also shown that the rs2910164 modifies clinical outcome of female patients with the follicular variant of papillary thyroid carcinoma. Due to insufficient patient number, the effect of the polymorphism on the clinical outcome of male patients could not be determined. Interestingly, we observed no impact of the SNP on mortality of patients with the classical variant of PTC, which is believed to be less aggressive compared to follicular variant [16]. Still, the noninvasive variant of fvPTC is considered to be the low-risk disease, and is managed by lobectomy only with no further radioactive iodine therapy. However, our study showed that the rs2910164 CC genotype was independently associated with higher mortality in groups of patients considered as low-risk, i.e., with a low stage of the disease, no extrathyroidal invasion and no distant metastases. The data suggest that the allelic status of rs2910164, similarly to the previously described rs966423 [1], can be used to identify high-risk female patients in groups that would otherwise be treated more conservatively. Contrary to somatic changes putatively associated with mortality, the status of germline variants is known before the surgery, and might be used as pre-treatment marker in management of mortality risk by means of modification of therapy.

The influence of rs2910164 on the transcriptome of PTC tumors was revealed in previous studies, analyzing gene expression levels in PTC tumors from patients harboring the GG or GC genotype in miR-146a-3p. The study revealed that the presence of the SNP alters the expression of genes implicated in many processes regulating cell division and apoptosis [10,11]. The presence of the C allele resulted in exaggeration of the DNA-damage response pathway and of the anti-apoptotic NF-kappa-B cascade, indicating the pro-tumorigenic role and possible radioresistance role of the SNP.

Why the association between rs2910164 CC genotype and higher mortality rate is found in patients with fvPTC but not conventional PTC is unknown, but could potentially result from different pathways which are disrupted in the two PTC variants. As indicated by molecular studies, follicular and classical variants of PTC differ by the expression of 1689 microRNA isoforms and 8271 gene transcripts [17] that could be the up- or downstream effectors of the SNP. The importance of rs2910164 is underlined by the presence of somatic mutations in tumors. We showed that 16.1% of analyzed specimens undergo somatic mutation within miR-146a-3p, and 70% of mutations towards the risk “C” allele creating the heterozygosity. Of note, heterozygosity is the only state when a single cell simultaneously expresses miR-146a-3p^G and miR-146a-3p^C, both down-regulating the expression and function of NIS. The lack of expression of NIS in thyroid tumor cells is an important factor contributing to radioiodine refractory disease. The impact of somatic mutation on the clinical outcome of PTC patients could not be evaluated due to small sample number.

A number of studies have shown that the expression of NIS can be re-induced by retinoic acid (RA) and such induction restores the ability of cells to concentrate radioactive iodide [18,19,20], but the effect is limited by reduced levels of RARB. Interestingly, during our studies we noticed that both miR-146a-3p^G and miR-146a-3p^C directly bind also *RARB* gene, however, since *RARB* levels are not detectable in MCF7 cells, further functional studies of both genes, *RARB* and *NIS* together, were not possible. Nevertheless, our data and already published studies [21] suggest that both NIS and RARB are regulated by the whole family of miR-146 (miR-146a and miR-146b) making the family of miR-146 an important therapeutic and prognostic target in PTC.

The main limitation of the study is lack of validation cohort and bigger group of male patients. Unfortunately, the study of the overall survival in cancers with low-mortality requires especially large cohort with long follow-up, and such cohort of PTC patients, especially fvPTC subtype and male gender, is not available. Other limitation includes relatively small number of analyzed tissue samples that does not allow for statistical analysis of the patients’ clinical outcome.

To our knowledge, the rs2910164 is the only genetic marker allowing for stratification of fvPTC female patients prior to the surgery, what might be particularly important for patients previously considered as low-risk. Although its clinical utility requires further large studies, this and the previously described rs966423 *DIRC3* variant (relevant for all differentiated thyroid cancer patient) are promising non-invasive marker of the clinical outcome of PTC patients.

## 4. Materials and Methods

### 4.1. Study Patients

Patients with PTC (*n* = 2441; 2163 women and 278 men; median age at diagnosis 48 years, SD ± 14), including patients with classical variant (2082 cases) and follicular variant PTC (fvPTC, 359 cases, including 315 females), were recruited at the Maria Skłodowska-Curie Memorial Cancer Center-Institute of Oncology, Poland. Diagnosis was confirmed through histopathological examination performed by 2 independent pathologists and TNM and clinical staging was defined on the basis of the 2010 American Joint Commission on Cancer staging system. Due to the fact, that the pathological examination was performed prior to the issue of the current 2017 WHO classification, the noninvasive follicular thyroid neoplasm with papillary-like nuclear features has not been evaluated. Genotyping of the rs2910164 in blood-derived DNA of PTC patient was performed using the Sequenom© and Taqman© technology, as described previously [22] and clinicopathological data were collected during medical examinations as well as from medical records. The median follow–up time of the patients was 10.25 years. The analysis was based on the overall mortality of the patients assuming that all the genotypes had an equal chance of dying due to non-PTC reasons. Also, overall mortality is free from the biases, including incorrect identification of the cause of death [23].

### 4.2. Somatic SNP Genotyping

The genotyping of miR-146a in cancer tissue was performed in DNA from fresh-frozen paraffin-embedded blocks (FFPE) of resected thyroid tissue obtained from 186 PTC patients (Table S3). The SNP was genotyped in blood-derived DNA and FFPE-derived DNA from the same patients using the Taqman© SNP genotyping assay (cat. no. 4351376 Life Technologies, Waltham, Massachusetts, USA) in standard conditions. All reactions were performed on a LightCycler^®^ 480 Instrument (Roche, Basel, Switzerland). All somatic mutations identified were further confirmed in a Sanger sequencing using primers provided in Table S2.

### 4.3. In Silico miR Analysis

The binding sites for miR-146a in 3′UTR regions of *NIS* were identified using TargetScan 6.1 (available online: http://www.targetscan.org/), miRanda (available online: http://www.microrna.org/), miRecords (available online: http://mirecords.biolead.org) and PITA (available online: http://genie.weizmann.ac.il).

### 4.4. Gene Expression Quantification

Gene expression quantification was performed using the TaqMan probes for *NIS* and the *HPRT* reference gene (Table S2). All quantifications were performed on a LightCycler^®^ 480 Instrument (Roche).

### 4.5. Direct miR and NIS Binding Luciferase Assay

For the analysis of interaction between the microRNAs and *NIS*, a plasmid containing hNIS-3′UTR cloned downstream of the *Firefly* luciferase and a *Renilla* luciferase gene as an internal control (pEZX-MT01, ID-HmiT017390-MT01) was purchased from GeneCopoeia (Rockville, MD, USA). Cervical cancer cell lines (HeLa, ATCC^®^ CCL-2^TM^) with low endogenous expression of *NIS* and miRs of interest were cultured in 90% High glucose Dulbecco’s Modified Eagle Medium (DMEM) and 10% Fetal Bovine Serum (FBS) (Life Technologies, Waltham, MA, USA), and subsequently transfected with abovementioned hNIS-plasmid and microRNA expression plasmid (700 ng pCDNA3-miR-146a-5p-3p^C, 500 ng pcDNA3-miR-146a-5p-3p^G) or the same amount of pcDNA3 negative control using Fugene HD (Promega, Madison, WI, USA).

Luciferase activity was measured by the Glomax-Multi Detection System (Promega Corporation) using Dual-Luciferase Reporter 1000 Assay System (Promega Corporation); *Firefly* luciferase activity was normalized to *Renilla* luciferase activity.

### 4.6. MicroRNA miR-146a Inhibition

Inhibition of microRNAs was attained with the use of specific microRNA sponge plasmids [24]. For inhibition of miR-146a-5p, sequence complementary to the miR was cloned into the pGL3 expression plasmid as previously described [21]. For inhibition of all miR-146a-5p, miR-146a-3p^G variant, miR-146a-3p^C variant, an 80-nt long oligonucleotide containing sequences complementary to the microRNA was synthesized, amplified with specific primers containing restriction sites for PstI and KpnI, and cloned into the pGL3 control vector to yield pGL3-sponge-miR-146a-5p-3p^G-3p^C. Negative control was cloned accordingly (Table S2).

There is no immortalized human thyroid cell-line that expresses endogenous NIS. To investigate the effect of inhibition of miRs on endogenous *NIS*, we chose MCF7 (human breast adenocarcinoma) cell line. It is well established that the expression and function of NIS can be induced in MCF7 cell line by trans-retinoid acid/hydrocortisone (tRA/H) [18,25]. MCF-7 cells (ATCC^®^ HTB-22^TM^) were cultured in 45% DMEM, 45% F-12 and 10% FBS (Life Technologies). MCF-7 cells were treated with 1 μM tRA/H [2] (Sigma-Aldrich, Saint Louis, MO, USA) for 24 h to induce *NIS* expression. Induction with tRA/H was performed in the presence of 5% FBS instead of 10% regular FBS. Subsequently cells were transfected with a 1000 ng of a microRNA sponge plasmid (pGL3-sponge-miR-146a-5p, pGL3-sponge negative control, or pGL3-sponge-miR-146a-5p-3p^G-3p^C using Fugene HD (Promega, Madison, WI, USA). Total RNA was extracted from cell cultures, and *NIS* mRNA levels were measured using real-time PCR.

### 4.7. RAIU Assay

MCF7 cells were transfected with microRNA sponge vector, and after 48 h, the transfected cells were incubated with 2 μCi Na^125^I in 5 μM non-radioactive NaI for 30 min at 37 °C with 5% CO_2_. Subsequently, cells were washed twice with cold Hank’s balanced salt solution (HBSS) and lysed with 95% ethanol for 20 min. The cell lysate was collected and radioactivity was counted by a scintillation counter. DNA was measured subsequently and fold-change values were calculated after adjusting CPM count per DNA amount and subtracting values for perchlorate-treated controls. Three independent experiments were performed in triplicates, and with additional well for perchlorate control.

### 4.8. Statistical Analyses

Patient mortality was calculated by dividing the number of deaths by the number of patients in each analyzed group. The Fisher exact test was used to compare mortality for each genotype in miR-146a-3p. Rates per person-year were calculated by dividing the number of deaths by the sum of total follow-up years, and Poisson regression was used to calculate the 95% confidence intervals. Kaplan-Meier survival curves and log-rank tests, and Cox proportional hazards regression analyses, censoring patients at the time of the database inquiry (3 January 2017), were used to compare survival by genotype. Proportional hazards regressions were adjusted by stratification for age at diagnosis (<50 years old, and ≥50 years old) and sex (if both female and male patients were analyzed) using the counting process formulation of Andersen and Gill. Because there were only 2 males in CC-carriers fvPTC patients (no deads) there was no possibility to obtain trustable result when include sex in the model and only female fvPTC patients were analyzed. All reported P values are 2-sided and significance was set at *p* < 0.05. All statistical analyses were performed using the R software package version 3.0.2 (Available online: http://www.r-project.org/).

STATISTICA 10 (StatSoft Inc.) and GraphPad Prism 5.03 for Windows (GraphPad Software, La Jolla, CA, USA) were used for statistical analyses of cell culture experiments. All experiments were performed in at least three independent trials with three replicates for each experimental group within each trial. For luciferase assay, two sample t-test was performed for comparisons after taking the average of three replicates, and a *p*-value < 0.05 was considered significant.

## Figures and Tables

**Table 1:** The association of rs2910164 with various clinicopathological factors in follicular variant of papillary thyroid carcinoma (fvPTC) patients. n/a –not applicable.

Mortality, No./Total (%)				Deaths per 1000 Person-Years (95% CI)			Unadjusted		Adjusted	
Category	Overall	miR146a-GG/CG	miR146a-CC	Person-Years Follow-Up	miR146a-GG/CG	miR146a-CC	Log-Rank *p*-Value	HR (95%CI)	Log-Rank *p*-Value	HR (95% CI)
Female patients	19/315 (6)	17/308 (5.5)	2/7 (28.6)	3414.58 (3347.25 + 67.33)	5.08 (2.96–8.13)	29.7 (3.6–107.3)	0.003	7.03 (1.58–31.19)	0.006	6.21 (1.38–27.93)
Age										
<50 year	3/203 (1.5)	3/200 (1.5)	0/3 (0)	2277.58 (2240.08 + 37.5)	1.34 (0.28–3.91)	n/a	n/a	n/a	n/a	n/a
≥50 year	16/112 (14.3)	14/108 (13)	2/4 (50)	1137 (1107.17 + 29.83)	12.64 (6.91–21.22)	67.05 (8.12–242.2)	0.002	7.85 (1.68–36.55)	0.002	7.85 (1.68–36.55)
pT										
pT1 + pT2	9/241 (3.7)	8/237 (3.4)	1/4 (25)	2513.25 (2479.08 + 34.17)	3.23 (1.39–6.36)	29.27 (0.74–163.06)	0.002	13.62 (1.57–118.11)	<0.001	25.05 (2.23–280.9)
pT3 + pT4	9/65 (13.8)	8/62 (12.9)	1/3 (33.3)	737.42 (704.25 + 33.17)	11.36 (4.9–22.38)	30.15 (0.76–167.97)	0.183	3.83 (0.46–32.26)	0.642	1.66 (0.19–14.25)
pN										
N0	17/245 (6.9)	16/240 (6.7)	1/5 (20)	2607.42 (2562.42 + 45)	6.24 (3.57–10.14)	22.22 (0.56–123.81)	0.143	4.09 (0.53–31.52)	0.143	4.09 (0.53–31.52)
N1a	1/30 (3.3)	0/29 (0)	1/1 (100)	381.67 (369.42 + 12.25)	0 (0–9.99)	81.63 (2.07–454.83)	n/a	n/a	n/a	n/a
N1b	1/26 (3.8)	1/25 (4)	0/1 (0)	274.83 (264.75 + 10.08)	3.78 (0.1–21.04)	0 (0–365.96)	n/a	n/a	n/a	n/a
pN										
N0 + N1a	18/275 (6.5)	16/269 (5.9)	2/6 (33.3)	2989.08 (2931.83 + 57.25)	5.46 (3.12–8.86)	34.93 (4.23–126.2)	0.002	7.38 (1.65–33.11)	0.002	7.84 (1.72–35.69)
N1b	1/26 (3.8)	1/25 (4)	0/1 (0)	274.83 (264.75 + 10.08)	3.78 (0.1–21.04)	0 (0–365.96)	n/a	n/a	n/a	n/a
capsule										
N	6/193 (3.1)	5/189 (2.6)	1/4 (25)	2049.33 (2015.17 + 34.17)	2.48 (0.81–5.79)	29.27 (0.74–163.06)	0.016	8.95 (1.03–77.62)	0.021	9.03 (0.94–87.18)
T0 + T1	13/104 (12.5)	12/101 (11.9)	1/3 (33.3)	1149.92 (1116.75 + 33.17)	10.75 (5.55–18.77)	30.15 (0.76–167.97)	0.11	4.73 (0.58–38.59)	0.282	3 (0.37–24.7)
angioinvasion										
N	12/228 (5.3)	12/224 (5.4)	0/4 (0)	2514.5 (2473.08 + 41.42)	4.85 (2.51–8.48)	0 (0–89.06)	n/a	n/a	n/a	n/a
T	5/56 (8.9)	4/54 (7.4)	1/2 (50)	583.17 (569.5 + 13.67)	7.02 (1.91–17.98)	73.15 (1.85–407.58)	n/a	n/a	n/a	n/a
pM										
M0	18/292 (6.2)	16/285 (5.6)	2/7 (28.6)	3191.25 (3123.92 + 67.33)	5.12 (2.93–8.32)	29.7 (3.6–107.3)	0.003	7.04 (1.57–31.54)	0.005	6.5 (1.43–29.52)
M1	1/10 (10)	1/10 (10)	0/0 (NaN)	89.5 (89.5 + 0)	11.17 (0.28–62.25)	n/a	n/a	n/a	n/a	n/a
multifocality										
single	14/207 (6.8)	13/203 (6.4)	1/4 (25)	2254.33 (2207.58 + 46.75)	5.89 (3.14–10.07)	21.39 (0.54–119.18)	0.169	3.79 (0.49–29.25)	0.232	3.26 (0.42–25.41)
multifocality	5/95 (5.3)	4/92 (4.3)	1/3 (33.3)	990.42 (969.83 + 20.58)	4.12 (1.12–10.56)	48.59 (1.23–270.73)	n/a	n/a	n/a	n/a
Stage										
I + II	9/214 (4.2)	8/210 (3.8)	1/4 (25)	2218.83 (2184.67 + 34.17)	3.66 (1.58–7.22)	29.27 (0.74–163.06)	0.004	12.01 (1.38–104.19)	<0.001	22.72 (2.04–253.66)
III + IV	9/95 (9.5)	8/92 (8.7)	1/3 (33.3)	1100.33 (1067.17 + 33.17)	7.5 (3.24–14.77)	30.15 (0.76–167.97)	0.073	5.63 (0.67–47.38)	0.522	1.99 (0.23–17.16)

## Supplementary Materials

Supplementary materials can be found at http://www.mdpi.com/1422-0067/19/3/655/s1.
